# SARS-CoV-2 in a Mink Farm in Italy: Case Description, Molecular and Serological Diagnosis by Comparing Different Tests

**DOI:** 10.3390/v14081738

**Published:** 2022-08-08

**Authors:** Ana Moreno, Davide Lelli, Tiziana Trogu, Antonio Lavazza, Ilaria Barbieri, MariaBeatrice Boniotti, Giulia Pezzoni, Cristian Salogni, Stefano Giovannini, Giovanni Alborali, Silvia Bellini, Massimo Boldini, Marco Farioli, Luigi Ruocco, Olivia Bessi, Andrea Maroni Ponti, Ilaria Di Bartolo, Luca De Sabato, Gabriele Vaccari, Gabriele Belli, Alberto Margutti, Maurilio Giorgi

**Affiliations:** 1 Istituto Zooprofilattico Sperimentale della Lombardia e dell’Emilia Romagna, IZSLER, Via Bianchi, 9, 25124 Brescia, Italy; 2 Direzione Generale Welfare, Regione Lombardia, Piazza Città di Lombardia 1, 20124 Milano, Italy; 3 Direzione Generale Sanità Animale e Farmaci Veterinari, Ministero della Salute, Via Giorgio Ribotta, 5-00144 Roma, Italy; 4 Department of Food Safety, Nutrition and Veterinary Public Health, Istituto Superiore di Sanità, Viale Regina Elena 299, 00161 Rome, Italy; 5 Dipartimento di Prevenzione Veterinario, ATS Valpadana, Via Belgiardino, 6-26100 Cremona, Italy

**Keywords:** SARS-CoV-2 diagnosis, serology, phylogenetic analysis, mink farm, Italy

## Abstract

This study described a SARS-CoV-2 infection in minks on an Italian farm. Surveillance was performed based on clinical examination and a collection of 1879 swabs and 74 sera from dead and live animals. The farm was placed under surveillance for 4.5 months, from the end of July 2020, when a man working on the farm tested positive by RT-PCR, till mid-December 2020 when all the animals were sacrificed. Clinical examination revealed no clinical signs or increased mortality rates attributable to SARS-CoV-2, while diagnostic tests detected only four weak PCR-positive samples, but 100% of sera were positive for SARS-CoV-2 anti-S antibodies. The phylogenetic analysis of two SARS-CoV-2 sequences from two minks and the sequence of the worker showed that they belonged to different clades. It could be therefore assumed that two distinct introductions of the virus occurred on the farm, and that the first introduction probably occurred before the start of the surveillance period. From the data collected, and especially from the detection of specific antibodies through the combination of different tests, it can be postulated that syndromic surveillance combined with genome detection by PCR may not be sufficient to achieve a diagnosis in asymptomatic animals. In particular, the serological approach, especially when using tests directed towards the S protein, may be useful for improving the traceability of virus circulation in similar environments.

## 1. Introduction

An emerging coronavirus, severe acute respiratory syndrome coronavirus 2 (SARS-CoV-2), causing COVID-19 disease, was identified in humans in Wuhan City in December 2019. The global spread of SARS-CoV-2 has been so extensive that the World Health Organization (WHO) declared COVID-19 a global pandemic on 11 March 2020. Since it was first reported in December 2019, SARS-CoV-2 has affected more than 257 million people, causing more than 5 million deaths worldwide [1]. SARS-CoV-2 is presumed to have emerged from an animal source and then spilled over into humans, with spread subsequently ensured by human-to-human infection [2]. The origin and route of introduction into the human population remain unclear [3]. However, the fact that some coronaviruses closely related to SARS-CoV-2 have been detected in *Rhinolophus* bats suggested that bats were the natural hosts of the virus [4]. The most likely hypothesis for the origin of SARS-CoV-2 may be that it began in bats and reached humans via a complicated pathway involving one or more intermediate animals that have not yet been definitively identified [2].

Several animals, such as domestic dogs and cats, and felids in zoos, have also been naturally infected with SARS-CoV-2 from humans [5]. Minks were the first farmed animals to be infected with SARS-CoV-2, indicating an increased susceptibility of mustelids to the virus [6]. Farmed minks for fur production were found to be infected after exposure to infected humans in many countries (Canada, Denmark, France, Greece, Italy, Lithuania, Netherlands, Spain, Sweden, United States) [7,8,9]. To date, 358 outbreaks in mink have been reported, including 20 cases in North America and 338 in Europe [9]. The most affected countries are Denmark [10] and the Netherlands [3]. Respiratory and, less frequently, gastrointestinal clinical signs were observed in affected animals; however, in most cases, the only sign of virus circulation was a slight increase in animal mortality [6]. In most of the affected farms, the infection was probably introduced through human–mink transmission due to SARS-CoV-2 infection in workers. However, minks may also act as a reservoir of SARS-CoV-2, transmitting the virus among themselves, with the risk of virus spillback from mink to humans. It has also been established that human-to-mink and mink-to-human transmission may occur [3,10,11].

Two SARS-CoV-2-positive farms of minks (*Neovison vison*) were detected in Italy, both characterised by serological positivity in the absence of symptoms in the animals [12]. The aim of this study was to provide an in-depth description of the SARS-CoV-2 infection in the first Italian farm that incurred positive results, considering anamnestic description, diagnostic tests for the detection of SARS-CoV-2 genome and antibodies, complete genome sequencing, and phylogenetic analyses. Furthermore, the diagnostic capabilities of different commercial and in-house serological tests for the serological analysis of mink serum samples were preliminary assessed.

## 2. Materials and Methods

### 2.1. Case Description and Sampling

A farm with 28,000 minks in the province of Cremona (Northern Italy) was placed under observation for 4.5 months from the end of July to mid-December 2020. SARS-CoV-2 surveillance based only on clinical visits began at the farm as required by note 0,011,120 of the Ministry of Health released on 14 May 2020. However, animal sampling was performed when a man working in the farm tested positive for SARS-CoV-2 by real-time reverse transcription polymerase chain reaction (RT-PCR) in July 2020. Clinical visits were performed during this period, and mink samples were collected at intervals of 1 d to 1 week. The surveillance period was completed when all animals were humanely sacrificed in December 2020 following the indication of the Ministry of Health (OM 21 November 2020) [13], which introduced the infection with SARS-CoV-2 among the notifiable diseases for which eradication was compulsory in the event of an outbreak.

**Figure 1:**
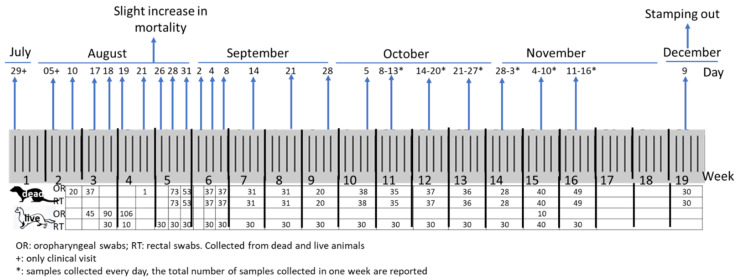
Timeline of and description of samples collected from the farm.

Clinical visits carried out periodically on the minks from the end of July to the end of August did not reveal any clinical signs or increased mortality. At the end of August, a slight increase in mortality and diarrhoea without respiratory signs was observed in some animals. Overall, a total of 1879 samples were divided into 593 oropharyngeal (OR) and 535 rectal (RT) swabs from dead animals, and 251 OR and 500 RT swabs from live animals were collected during the surveillance period. Moreover, 30 OR and RT swabs and 74 blood samples were collected from sacrificed animals during the culling procedures. Several tissues, including the lungs, liver, spleen, and intestine, were collected from symptomatic animals to perform diagnostic examinations. The description and timing of the samples collected from the minks on the farm are shown in Figure 1. In addition, oropharyngeal and nasal swabs collected on 1 August 2020 from the worker who tested positive for SARS-CoV-2 were processed for full genome sequencing.

### 2.2. Diagnostic Examinations

SARS-CoV-2 genome detection was performed using two real-time RT-PCRs targeting gene E [14] and gene N (OPTI SARS-CoV-2 RT-PCR kit, OPTIMedical, IDEXX, Hoofddorp, The Netherlands) [15]. Analysis for canine distemper virus (CDV) and influenza type A (IAV) were conducted using real-time RT-PCRs as previously described [16,17].

Bacteriological examinations were performed on the spleen, liver, and intestine tissues from animals with clinical signs by using standard bacteriological cultures [18]. For microorganism identification, matrix-assisted laser desorption ionisation-time of flight mass spectrometry (MALDI-TOF MS) and the ISO/TR 6579-3:2014 method [19] for serotyping of *Salmonella* serovars were used. Detection of *Clostridium* strains producing botulinum toxin of types A, B, E, F, C, D, CD, and DC was conducted using multiplex real-time PCR [20].

### 2.3. Serological Investigations

For serological analysis, several tests were performed to detect antibodies against different SARS-CoV-2 antigens.

Antibodies against nucleocapsid (N) protein were detected using enzyme-linked immunosorbent assay (ELISA) methods. First, a commercial indirect ELISA (IDscreen SARS-CoV-2 N IgG indirect ELISA, ID Vet, 34,790 Grabels, France) was performed as reported in the manufacturer’s guidelines, except for the use of a multispecies conjugate provided by the manufacturer. Then, two different double-antigen sandwich ELISAs were used to identify the total immunoglobulins against SARS-CoV-2 in animal sera: the Eradikit™ COVID19-Multispecies (In3 Diagnostics, 10095, Grugliasco (To), Italy) and an in-house IZSLER-double-antigen sandwich ELISA (DAS-N ELISA). Both methods were based on the recombinant N protein antigen coated onto the plate and the recombinant N protein-conjugated horseradish peroxidase (HRP). Eradikit™ COVID19-Multispecies ELISA was performed according to the manufacturer’s guidelines. The in-house double-antigen sandwich ELISA uses a recombinant SARS-CoV-2 N protein expressed in *E. coli*, purified by immobilised metal affinity chromatography, and conjugated with HRP as previously described [21]. Briefly, the unconjugated recombinant SARS-CoV-2 N protein (50 µL per well) was coated onto ELISA microplates and tested sera, diluted 1/2.5 in the diluent buffer in a final volume of 50 µL, was thereafter added to the coated plate and incubated for 60 min at room temperature. Subsequently, 50 µL/well of HRP-conjugated recombinant SARS-CoV-2 N protein was added. After incubation and washing, the 3,3′,5,5′-Tetramethylbenzidine (TMB) substrate (TMB supersensitive one component, Surmodics, Eden Prairie, MN, USA) was added, and the ELISA microplate was incubated in the dark at room temperature for 20 min. The results were expressed as a percentage of reactivity compared with the positive control included in each plate. For the expression of the results, the average optical density of the positive control was considered, and the S/P percentage was calculated for each sample by applying the following formula: % S/P: (optical density (OD) sample—average OD neg control/average OD pos control-average OD neg control) × 100.

Detection of Ab against the spike protein (S) was conducted using various assays including a virus neutralisation test (VNT), two different surrogate virus neutralisation tests (sVNT), and a double-antigen ELISA test. SARS-CoV-2 VNT was performed as described by Rijkers et al. [22], with few modifications. Briefly, sera were heat-inactivated (30 min, 56 °C) and tested in duplicates. Two-fold serial dilutions (starting at 1:10) of the sera were incubated with 100 TCID50 of the SARS-CoV-2 HCoV-19/Italy/310904/46/2020 strain (EPI_ISL_9011947) at 37 °C and 5% CO_2_ for 1 h at 37 °C in 96-well plates. Vero-E6 cells were added at a concentration of 2 × 10^4^ cells per well and incubated for 72 h at 37 °C with 5% CO_2_. The serum virus-neutralisation-titre (VNT50) was defined as the reciprocal value of the sample dilution that showed 50% protection against virus growth. Sera with titres ≥1/10 were considered positive for SARS-CoV-2 antibodies. Two SARS-CoV-2 sVNTs were included in this study. First, GenScript SARS-CoV-2 surrogate virus neutralisation (GenScript Biotech, Leiden, The Netherlands) was performed according to the manufacturer’s instructions. This method is a liquid-phase-blocking ELISA using human ACE-2 receptor protein (hACE2) coated on the plate and the receptor binding domain (RBD) from the S protein of SARS-CoV-2 conjugated HRP (HRP-RBD). Second, Proteogenix SARS-CoV-2 surrogate VNT (Proteogenix, Schiltigheim, France), based on the principle of competitive binding between serum Ab and HRP-ACE2 to the recombinant RBD of S protein coated onto the plate, was used. According to the manufacturer’s instructions, no pre-incubation of sera or the RBD antigen was required. Finally, the Wantai SARS-CoV-2 total antibody ELISA (Beijing Wantai Biological Pharmacy Enterprise, Beijing, China; catalogue number WS1096) was performed according to the manufacturer’s instructions.

In order to assess the specificity of the different serological tests employed, 44 mink sera were also collected from other Italian mink farms placed under SARS-CoV-2 surveillance. These animals showed no clinical symptoms, no epidemiological connection with SARS-CoV-2 positive cases and were always RT-PCR negative, and therefore, they were included as negative sera in the validation of the serological tests.

The comparison of the different serological methods was performed by taking into account the method and type of Abs detected. Diagnostic sensitivity, specificity values and Cohen’s kappa coefficient between each test and VNT assays were calculated.

### 2.4. Sequencing and Phylogenetic Analysis

RNAs were processed for sequencing using next-generation sequencing (NGS) technology at the Large Instrumentation and Core Facilities (FAST) service of Istituto Superiore di Sanità (Italy). The libraries for each sample, starting from the extracted RNA, were prepared using the Ion AmpliSeq method, based on the specific amplification of the viral target (ThermoFisher Scientific, Waltham, MA, USA), and libraries were sequenced using Ion S5 System technology (Thermo Fisher Scientific, Waltham, MA, USA) on an Ion 520 Chip. This method is based on a panel of primers designed to specifically amplify the complete genome of SARS-CoV-2. The obtained sequences were analysed using the Galaxy Aries online platform (https://aries.iss.it/root/login?redirect=%2F, accessed on 20 April 2021), on which the pipeline for the reconstruction of complete SARS-CoV-2 genomes, named SARS-CoV-2 RECoVERY, was implemented [23].

Phylogenetic and molecular analyses were performed using Nextclade Web 1.5.3 (https://clades.nextstrain.org/, accessed on 23 August 2021) (Ref, strain Wuhan/WH01/2019) [24]. A banded Smith–Waterman alignment with an affine gap penalty was performed. Nextclade assigns clades by placing the sequence in a tree representing the currently circulating SARS-CoV-2. The clade is then inferred from the point in the tree that the sequences attach to [25]. On the reference tree, clades were assigned using lists of clade-defining mutations via the augur workflow (https://github.com/nextstrain/ncov/blob/master/defaults/clades.tsv, accessed on 23 August 2021). The phylogenetic tree was visualised using the Auspice website [26]. To study the differences within the ACE2 interface with the Spike receptor binding domain (S-RBD) of SARS-CoV-2, the sequences of the human (huACE2) (genBank acc. N. Q9BYF1) and Neogale vison (MinkACE2) (genBank acc. N. QPL12211) ACE 2 receptors were compared using the Lasergene DNASTAR Megalign software, version 17.3.0.57.

## 3. Results

### 3.1. Diagnostic Examinations

**Figure 2:**
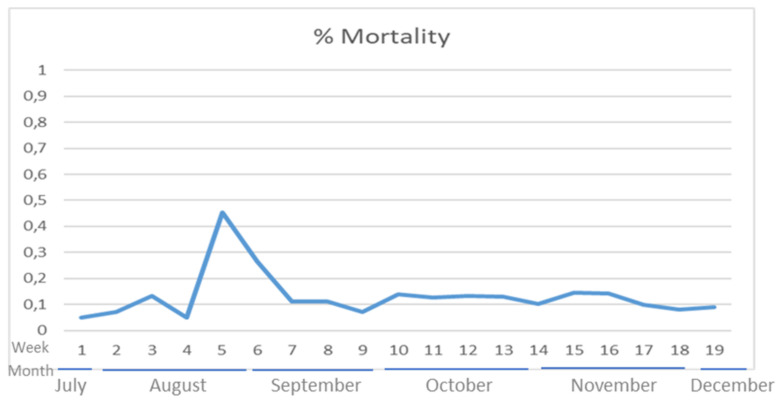
Percentage of weekly mortality on the farm during the observation period.

Detection of the SARS-CoV-2 genome was performed by performing real-time RT-PCR on both OR and RT swabs and tissues, showing only three weak-positive samples with high Cycle threshold (Ct) values close to the cut-off value (between 35 and 38 Ct values) and one doubtful sample (39 Ct value). The first positive result was observed from an OR swab collected from a dead animal during the first sampling on 10 August. Two additional low positive and doubtful samples were obtained from RT and OR swabs of the same animal on 18 and 19 August, respectively. The weekly mortality, constantly observed in the absence of clinical signs, was below 0.1%; however, an increase in mortality and diarrhoea was observed in late August with a peak of 0.45% (Figure 2). Thereafter, the viral surveillance was intensified with samples collected daily from dead animals and RT swabs collected once per week from live animals. All samples were negative for SARS-CoV-2 PCR until 29 October, when another RT swab was weakly positive.

Necropsy of animals showing clinical signs that had been submitted for diagnostic testing during this period revealed pulmonary congestion, splenomegaly, hepatic steatosis, and catarrhal enteritis. Histological examination was performed only on the best-preserved organs that did not undergo autolytic processes including lung-pulmonary oedema, spleen-marked extra medullary haematopoiesis, and the presence of a substantial infiltrate of plasma cells were observed, while intestine (small intestine) and kidney indicated no significant findings. Bacteriological tests were also performed on mink tissues, detecting a septicaemic *Streptococcus equi* in animals with clinical signs in two different collections on 28 and 30 August 2020. One *S. enteritidis* var. *enteritidis* was also detected in the faeces of animals which were collected on 30 August. Examinations for CDV and IAV genomes, and *Clostridium* sp. producing botulism toxins, were negative.

In September 2020, the mortality level returned to normal values, but sample collection and clinical surveillance continued weekly until 16 November 2020, when, after the last real-time RT-PCR positivity at the end of October, a decision was made to proceed with the culling of all minks on the farm. Culling operations were performed in the first two weeks of December 2020. Another set of samples, including OR and RT swabs from 30 animals, was collected on 9 December 2020, and all were negative.

### 3.2. Serological Investigations

Serological tests were performed on 74 blood samples collected on 9 December during the culling procedures on the positive farm and on an additional 44 blood samples collected from other negative farms. The sera were analysed using a variety of tests capable of detecting several types of antibodies, providing a notable picture of the diagnostic performance of all kits for the serological diagnosis of SARS-CoV-2 in animal sera. A summary of the results obtained using the different serological assays including diagnostic sensitivity and specificity values and Cohen’s kappa coefficient between each test and VNT assays is provided in Table 1.

Antibodies against N protein were detected by applying three ELISA methods. The IDScreen indirect ELISA did not provide positive samples. The other two double-antigen ELISAs, which were specifically designed for animal sera, showed similar results, detecting 62/69 positive samples using the in-house IZSLER-double-antigen sandwich ELISA and 59/66 when implementing the Eradikit™ COVID19-Multispecies with a diagnostic sensitivity (Se) of 89.39% and 86.15%, respectively. However, these Se values were lower than that of the other methods for the detection of antibodies against S protein (Table 1).

Neutralising antibodies were successfully identified using the VNT assay, which showed positive results at different titers but above the 1/10 cutoff value only for all samples from the positive farm. The same positive results were obtained using one of the two sVNTs, the GenScript sVNT, which showed all sera from the infected farm as positive while the 44 sera collected from negative farms were negative. The other sVNT showed poor diagnostic performance as it did not identify any serum as positive. In addition, the Wantai SARS-CoV-2 total antibody ELISA revealed excellent diagnostic performance, providing positive results well above the cutoff value for all the sera from the infected farm and negative results for the sera from negative farms. For both the GenScript and Wantai tests, excellent diagnostic values for Se and Sp and a perfect agreement with VNT were found (Table 1). All sera collected from the mink farm with a diagnosis of SARS-CoV-2 by RT-PCR, subsequently confirmed by performing sequencing, were positive for VNT. Considering the origin of the sera and that the VNT is considered the gold standard for the detection of neutralising antibodies), the status of these sera was considered infected.

### 3.3. SARS-CoV-2 Genome Detection and Phylogenetic Analysis

Whole SARS-CoV-2 genome sequencing was attempted on the mink PCR-positive samples and positive samples collected from the worker. The analysis of the sequencing data of the human sample (collected on 8 August 2020) and two mink samples (collected on 17 August 2020 and 29 October 2020, respectively) showed satisfactory results, and the quality values are reported in Table 2. For the consensus sequence reconstruction, a minimum coverage of 30× was considered for each position, assigning to that position the majority variant (most represented nucleotide).

**Figure 3:**
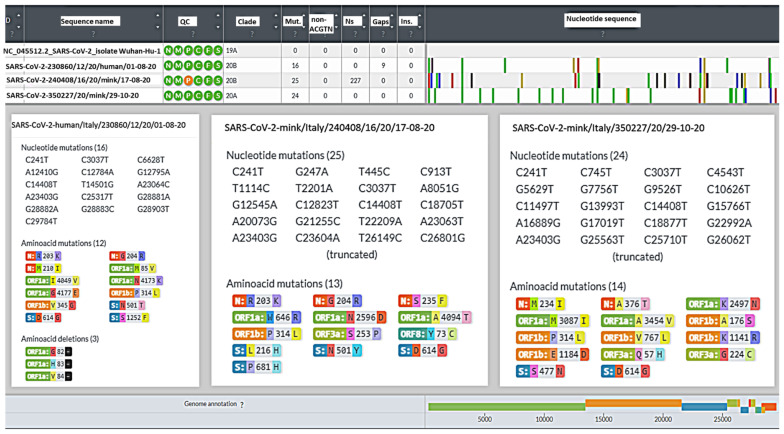
Summary of the results of Nextclade analysis. For each sequence nucleotide, amino acid mutations and deletions are reported.

**Figure 4:**
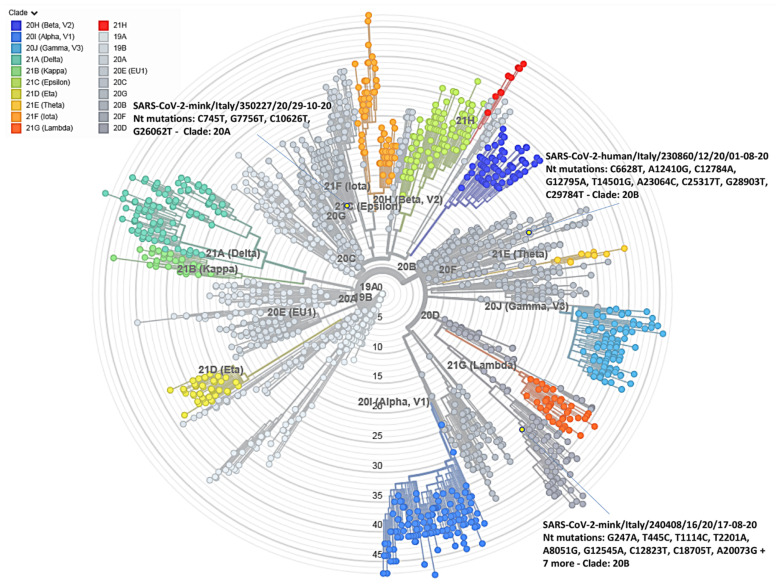
Maximum likelihood tree based on the complete genome SARS-CoV-2 and the three Italian sequences performed by Nextclade software. Groups are coloured according to the Nextclade assignment. Italian sequences are identified in yellow, and Nextclade assignment and nucleotide mutations are reported.

A whole-genome-based phylogenetic analysis showed that the Italian worker and mink SARS-CoV-2 sequences belonged to different clades, which corresponded to two distinct Nextstrain clades. The results of the Nextclade analysis, namely quality control evaluation, clade assignment, mutations, number of missing nucleotides, gaps, and insertions in the Italian sequences, as compared to the reference strain Wuhan 1, are shown in Figure 3. The SARS-CoV-2 sequences obtained from the worker and the mink sampled in August 2020 were assigned to Nextclade 20 B while the sequence from October 2020 was assigned to a different Nextclade, 20A (Figure 4). However, although the two August sequences belonged to the same Nextclade, different nucleotide and amino acid (aa) mutations were evident in the two sequences, suggesting a non-correlation between the worker and the first case detected in mink. The closer relationship for the three sequences was investigated by conducting an Audacity Instant search in GISAID (i) for the worker sequence, 102 related genomes for which the most frequent country was Portugal (27.5% of genomes), the most frequent lineage was B.1.1 (55.9% of genomes), and 81.6% of the related genomes were from samples collected between March 2020 and February 2021; (ii) for the mink sequence of August 2020, 41 related human genomes for which the most frequent country was United Kingdom (58.5% of genomes), the most frequent lineage was B.1 (87.8% of genomes), and 82.5% of the related genomes were from samples collected between December 2020 and March 2021; and (iii) for the mink sequence of October 2020, 94 related human genomes for which the most frequent country was Luxembourg (44.7% of genomes), the most frequent lineage was B.1.160 (97.9% of genomes), and 81.3% of the related genomes were from samples collected between October 2020 and December 2020. The maximum likelihood phylogenetic tree showing the three Italian sequences and nucleotide changes from Wuhan/WH01/2019 is illustrated in Figure 4.

The aa mutations observed in the S protein of the three Italian sequences compared to the reference sequence Wuhan/WH01/2019, are shown in Table 3. To investigate whether the aa mutations observed in the two mink sequences were also present in the sequences from humans in Italy, all human sequences available through the GISAID dataset from Italy collected in 2020 and 2021 were analysed for presence/absence of the mink aa mutations, and the results were expressed as the number of sequences with the mutations and %, as compared to the total number of sequences checked.

The sequence of MinkACE2 showed 137 aa different from that of huACE2, with them sharing an overall percentage of aa identity of 81.3%, but this similarity decreased to 64.5% in the three regions involved in the interaction with the S protein at positions 30–41, 82–93, and 353–358 [27]. A total of 17 S-RBD residues were in contact with 20 ACE2 residues [28]. Among the 20 ACE2 residues that interacted with the S-RBD, 13 were shared between human and mink ACE2 receptors (Figure S1). Of these, residues K31, Y41, and K353 required for the interaction were conserved, but MinkACE2 possessed D90 instead of N90, which could likely explain the difference in affinity with the S protein of SARS-CoV-2 between human and mink ACE2 receptors [27]. All the 17 S-RBD residues, except two (477 and 501), were conserved in the Italian sequences. Two mutations at position 501 were evidenced, N501T in the human sequence and N501Y in the mink sequence of August 2020, whereas S477N was present only in the mink sequence of October 2020. The amino acids expressed at positions 453 and 614 of the S protein had to be considered. The Y453F mutation, found in mink in Denmark and the Netherlands, was selected in mink after infection and could confer a selective advantage in mink-to-mink transmission. The F453 mutation was shown to be involved in a higher binding affinity to human ACE2 than Y [29]. The D614G mutation was previously reported as a variant that emerged in humans and was shown to confer a higher affinity for the ACE2 receptor [29]. The D614G mutation, but not Y453F, was observed in the three Italian sequences originating from the worker and the two minks. 

## 4. Discussion

The clinical case described presented a SARS-CoV-2 infection in a mink farm in Italy with an infection pattern and a clinical situation that were complex and difficult to interpret. The report of three low-titre real-time RT-PCR-positive swabs in the first sampling period from two animals collected at the beginning of August 2020, during surveillance and following a positive COVID-19 case in a farm-worker, led to the hypothesis of a SARS-CoV-2 infection on the farm. The PCR values were close to or just below the cutoff value, and the hypothesis of SARS-CoV-2 infection did not appear to be confirmed by subsequent negative real-time RT-PCR results obtained in the subsequent weeks, despite the large number of samples. The absence of clinical symptoms at the time appeared to be another factor that did not confirm the infection. Evidence of enteric symptoms and a slight increase in mortality one month after the start of the observation period led to an increase in surveillance activities focusing on dead animals with daily carcass sampling and swabbing. However, the diagnostic examinations of the carcasses revealed other aetiological agents, *Streptococcus equi* and *Salmonella enetritidis* var. *enetritidis*, as the potential cause of clinical signs. Sampling and surveillance continued periodically for a further two months without the detection of any positive samples. However, at the end of October 2020, another low-titre positive sample was detected from a rectal swab without observing any clinical signs. The detection of a third positive animal, albeit at low titre, together with the approval of the OM 21 November 2020, which introduced SARS-CoV-2 infection among the notifiable diseases for which eradication was mandatory in the event of an outbreak, resulted in the decision to cull all the animals present on the farm as soon as possible. This decision provided the opportunity to acquire sera for serological investigations. Quite surprisingly, we found a high percentage of positive serological samples in sera collected at the time of culling, as evidenced by 100% positive results with the VNT, the GeneScript surrogate VNT and the Wantai SARS-CoV-2 Ab ELISA. This high serological prevalence in apparently healthy animals suggested a widespread diffusion of the virus within the farm, which did not seem likely given the results observed with real-time RT-PCR.

These findings highlighted that surveillance based on genome detection alone may not be sufficient to make the diagnosis in asymptomatic animals. In addition, reports of increased mortality not related to SARS-CoV-2 but to other aetiological agents may further complicate such a situation as they can lead to a focus on the sampling of dead animals and thus make diagnosis even more difficult. Serology can be extremely useful in these cases to improve investigations of the circulation of the virus in the herd.

The use of various serological tests performed on mink blood samples yielded data on their ability to identify SARS-CoV-2 positive animals, both indirectly and potentially retrospectively. However, it should be considered that most commercial serology kits were developed and validated to test human sera, and little is known of their diagnostic performance with animal sera. Therefore, to verify their suitability as diagnostic tools in the diagnosis of SARS-CoV-2 in animals, we used and compared the performances of a range of serological tests that could detect antibodies to different antigens such as N protein or the S-RBD, and several types of antibodies such as total antibodies (Ig M, Ig G and IgA), Ig G only, and neutralising antibodies. The serological methods used included an indirect ELISA detecting IgG and three double-antigen ELISAs identifying total antibodies. Neutralising antibodies capable of interfering with the binding of the RBD of the S protein to the human cell surface receptor ACE2 could be detected by either VNT or sVNT, depending on whether live viruses were used for VNT or recombinant proteins for sVNT. The serological results obtained showed a better antibody response to the RBD of the S protein than in the N protein, as detected by the higher sensitivity of diagnostic tests capable of detecting anti-RBD antibodies than those capable of detecting anti-N antibodies. These results were in line with those of other authors who observed a higher sensitivity in methods detecting neutralising antibodies than for anti-N antibodies in pigs after experimental infection [30], and in cats and lions after natural infection [21,31].

Regarding the ELISA tests for the detection of anti-N antibodies, the best diagnostic performance was observed when using the two double-antigen ELISA tests (IZSLER and Eradikit) that were developed and validated for animal sera with excellent agreement (K > 0.8), as compared to VNT but a lower sensitivity (89,39 and 86,15%, respectively) than VNT, which showed 100% sensitivity. Additionally, all methods used to detect antibodies to RBD produced better results than those for anti-N antibodies, with the exception of an sVNT test. Of the two surrogate kits, one (sVNT geneScript) provided good performance (Se 100% and Sp 100%) with mink sera and excellent agreement (K = 1) with VNT. Similar results were previously published for human sera [32] and for human and animal sera, although with slightly lower sensitivity in the latter [33]. The other sVNT (Proteogenix) was not able to identify mink sera as positive. Although both were sVNT kits, the procedures differed. The geneScript kit was characterised by the recombinant human ACE2 protein (rhu-ACE2) adsorbed to the plate and the recombinant RBD-conjugated HRP (RBD-HRP). The protocol involved the pre-incubation of serum samples with RBD-HRP to facilitate the binding of the antibodies present in the sample to the RBD. The mixture was then added to the coated plate. Unbound RBD-HRP were captured on the plate while circulating RBD-HRP neutralising antibody complexes remained in the supernatant and were removed during washing. The Proteogenix kit was based on the recombinant RBD protein coated on the plate and involved the addition of the serum sample mix and the rhu-ACE2-conjugated HRP to the coated plate, placing any antibodies present in the serum and the rhuACE2-HRP under the same conditions for binding to the RBD. Direct competition for binding to the RBD between the antibodies in the sample and rhu-ACE2 in the absence of prior pre-incubation between the antibodies and the RBD probably resulted in a decrease in the sensitivity of the method. Notably, this method was validated for human sera; therefore, human antibodies likely bind to the RBD of SARS-CoV-2, a human virus, better than mink antibodies. The ELISA Wantai also provided very good diagnostic performances (Se 100% and Sp 100%) and excellent agreement (K = 1) with VNT, but the larger amount of serum required, as compared to the other methods (100 microlitres), could be an issue when analysing sera from certain animal species for which the collection of large volumes of serum would be difficult.

In addition to the serological tests used in our study, other assays such as luciferase immunoprecipitation systems (LIP-S) were validated in mink and other experimentally infected animals [34], demonstrating the suitability of the SARS-CoV-2-LIPS test for the detection of antibodies against the S protein in the serosurveillance of SARS-CoV-2 infection in a range of animal species. The LIPS-S test showed a better discriminatory power between positive and negative samples than the anti-N antibody test. These results demonstrated a higher reliability for serological methods to detect antibodies to the S protein than to the N protein in animal sera.

To support the etiological diagnosis of SARS-CoV-2, the genomic approach was used to better explain the epidemiological pattern of the observed case and to try to establish its origin and inception. Accordingly, the complete genome sequence of the three positive samples obtained, respectively, from the worker on the farm and the infected mink in August 2020 (mink1) and the last infected mink (mink2) in October 2020 were obtained using an NGS protocol. Unexpectedly, despite the large number of swabs analysed, only three minks were found positive by PCR, and complete sequencing was only obtained for samples from two animals. The analysis of the complete genome sequences of the three samples showed several nucleotides and amino acid differences from the Wuhan reference sequence (Figure 3). Several changes were also found that differentiated the three Italian sequences (one from the worker and two from the minks), evidenced by both their Nextclade assignments (20B for human and mink1 and 20A for mink2) and their varied positions in the phylogenetic tree. Nextclade 20A emerged from 19A, dominating the European outbreak in March, and has since spread globally; additionally, Nextclade 20B is a large, genetically distinct 20A subclade that emerged in early 2020. Notably, all sequences had the 614G variant, but none of them possessed the aa mutations described in the mink variant (cluster 5) described in Denmark, characterised by aa changes in the S protein, including three substitutions (Y453F, I692V, and M1229I) and a loss of two aa residues, 69 and 70 (ΔH69/V70) [35]. Other substitutions identified in SARS-CoV-2 isolates derived from mink in other countries were two aa substitutions (G261D, A262S) in the N-terminal domain of the S protein and four (L452M, Y453F, F486L, N501T) in the RBD [36]. One other interesting mutation identified in in the Italian mink sequence of August was the P681H mutation associated with enhanced protein S cleavage [37].

Overall, the results obtained from the phylogenetic analysis did not allow us to establish a connection between the worker and the sequences detected in the two minks. It is therefore suggested that independent events could have caused infection in the worker and the minks and that the virus that infected the minks in August 2020 (mink1) was probably introduced into the farm before the start of the observation period and not as a consequence of the infected worker. In addition, the differences between the August (mink1) and October (mink2) sequences suggested that there were two different viral introductions into the farm and that the mink population was still susceptible to infection with a different strain of SARS-CoV-2, even after infection. A similar possibility was described by other authors who hypothesised the likelihood of reinfection in seropositive mink after an initial SARS-CoV-2 infection, followed by recovery [38]. In our case, however, the absence of serological tests conducted during the observation period did not allow us to determine the serological prevalence on the farm after the first viral introduction.

## 5. Conclusions

The high seroprevalence of SARS-CoV-2, revealed by the combined use of different serological methods, confirmed virus circulation in mink farms without specific clinical signs or a clear increase in mortality, even with a very low number of real-time RT-PCR positive samples. These results underlined the extreme usefulness of serological tests, especially those aimed at detecting antibodies to the S protein which confirm SARS-CoV-2 exposure of minks and improve the investigation of virus circulation in mink farms. A correlation between the human and mink sequences was not demonstrated by sequencing; therefore, considering all the findings, at least two distinct viral introductions into the farm could be assumed.

## Figures and Tables

**Table 1:** Serological results obtained using various tests. Positive results are highlighted in grey. NT: not tested due to insufficient quantity. Serum status is identified with two colours in the column of the number of samples: grey, positive; white, negative. The diagnostic accuracy of each test was calculated with the diagnostic test 2 *×* 2 table according to serum status using MedCalc software (https://www.medcalc.org, version 20.106, accessed on 30 May 2022) (Se: sensitivity; Sp: specificity).

n.samples	IDScreen SARS-CoV-2N IgGIndirect Multispecies Conjugate	ERADIKIT™ COVID19-Multispecies	Double-Antigen N ELISA IZSLER	Wantai SARS-CoV-2 Ab ELISA	GenScript Surrogate VNT	Proteogenix SARS-CoV-2 Surrogate VNT	VNT (Rijkers et al., 2020)
CUT-Off	S/P% ≥ 40	S/P% ≥ 20	S/P% ≥ 10	OD > 1000	% Inhibition > 30	% Inhibition	VNT50 ≥ 1/10
1	<0.3	8.0	11.4	4549	95	0	1/80
2	<0.3	100.3	40.5	4257	96	0	1/80
3	<0.3	200.7	37.2	4923	96	6	1/160
4	<0.3	287.9	107.0	4584	95	0	1/160
5	<0.3	155.2	73.6	4051	96	0	1/160
6	<0.3	NT	NT	4217	95	NT	1/160
7	<0.3	39.7	28.1	4209	96	1	1/160
8	<0.3	120.2	64.5	4157	96	0	1/80
9	<0.3	190.9	61.2	3481	78	0	1/80
10	<0.3	125.3	57.4	4171	95	10	1/80
11	<0.3	103.7	36.0	4305	95	0	1/40
12	<0.3	247.8	64.2	4104	94	23	1/160
13	<0.3	279.3	83.5	4462	95	0	1/320
14	<0.3	17.0	14.6	4310	48	0	1/10
15	<0.3	NT	114.4	4438	95	NT	1/40
16	<0.3	167.0	49.5	4500	95	0	1/80
17	<0.3	106.5	35.9	5064	96	0	1/80
18	<0.3	51.6	23.1	4416	95	0	1/160
19	<0.3	108.3	38.0	4365	96	0	160
20	<0.3	160.0	43.2	4247	95	0	1/160
21	<0.3	17.7	2.2	4954	81	0	1/40
22	<0.3	270.5	47.1	4688	78	0	1/10
23	<0.3	292.4	87.1	6068	96	16	1/160
24	<0.3	194.8	69.1	4415	49	0	1/10
25	<0.3	210.6	57.2	5290	96	0	1/160
26	<0.3	93.0	73.6	4512	94	0	1/160
27	<0.3	39.4	73.3	5027	96	0	1/80
28	<0.3	94.0	33.8	5165	94	0	1/160
29	<0.3	177.2	53.6	6016	91	0	1/80
30	<0.3	178.3	44.2	4840	96	0	1/80
31	<0.3	8.8	15.1	4694	94	0	1/80
32	<0.3	33.0	18.2	4472	96	0	1/160
33	<0.3	14.4	0.21	4870	67	0	1/10
34	<0.3	3.1	0.2	5171	95	0	1/80
35	<0.3	99.1	27.0	5171	89	0	1/40
36	<0.3	137.9	50.2	4460	89	0	1/160
37	<0.3	27.4	6.12	4419	44	0	1/10
39	<0.3	NT	NT	4875	95	NT	1/160
40	<0.3	135.4	24.3	5419	95	0	1/80
41	<0.3	1.8	12.2	6021	95	3	1/160
42	<0.3	54.5	4	4817	95	0	1/40
43	<0.3	238.4	96.5	5118	95	0	1/80
45	<0.3	45.7	17.4	4907	96	6	1/40
46	<0.3	277.1	112.5	4845	95	11	1/640
47	<0.3	13.4	0.66	6068	80	0	1/40
48	<0.3	234.1	65.3	5222	94	0	1/80
49	<0.3	31.5	7.7	4892	94	0	1/80
50	<0.3	38.8	17.4	4590	95	17	1/320
51	<0.3	275.4	53	4687	95	0	1/160
52	<0.3	NT	NT	4536	94	NT	NT
53	<0.3	158.2	63.7	4477	94	0	1/80
54	<0.3	26.8	14.8	4477	95	0	1/160
55	<0.3	79.3	20.3	4521	96	11	1/160
56	<0.3	164.9	90.8	4337	94	0	1/160
57	<0.3	124.7	43.6	4932	95	0	1/640
58	<0.3	23.3	15.3	5601	95	0	1/320
59	<0.3	NT	NT	4615	94	NT	1/20
60	<0.3	36.2	20.0	4716	88	0	1/40
61	<0.3	NT	NT	6078	95	NT	NT
62	<0.3	52.2	30.0	4902	95	0	1/160
63	<0.3	142.7	44.2	4692	51	0	1/20
64	<0.3	228.6	91.0	4838	96	0	1/160
65	<0.3	64.1	15.1	4537	88	0	1/40
66	<0.3	176.3	92.3	4509	95	0	1/160
67	<0.3	26.0	19.5	4434	95	0	1/160
68	<0.3	15.9	19.2	4201	94	0	1/80
69	<0.3	84.1	25.2	4652	95	0	1/160
70	<0.3	123.1	50.2	4236	95	0	1/160
71	<0.3	29.5	13.5	4270	89	0	1/40
72	<0.3	245.4	58.8	4115	94	0	1/80
73	<0.3	115.2	86.9	3995	96	0	1/160
74	<0.3	NT	NT	3999	89	NT	1/40
n.samples	IDScreen SARS-CoV-2N IgGIndirect Multispecies conjugate	ERADIKIT™ COVID19-Multispecies	Double-Antigen N ELISA IZSLER	Wantai SARS-CoV-2 Ab ELISA	GenScript Surrogate VNT	Proteogenix SARS-CoV-2 surrogate VNT	VNT (Rijkers et al., 2020)
CUT-off	S/P% ≥ 40	S/P% ≥ 20	S/P% ≥ 10	OD > 1000	% inhibition > 30	% inhibition	VNT50 ≥ 1/10
75	<0.3	11.7	4.9	98	11	NT	<1/5
76	<0.3	12.6	3.8	83	0	NT	<1/5
77	<0.3	10.0	5	74	9	NT	<1/5
78	<0.3	13.8	4.6	77	9	NT	<1/5
79	<0.3	13.6	3.8	35	5	NT	<1/5
80	<0.3	6.0	4.1	87	0	NT	<1/5
81	<0.3	5.8	4.5	84	0	NT	<1/5
82	<0.3	6.8	4.3	159	2	NT	<1/5
83	<0.3	3.2	4.2	139	4	NT	<1/5
84	<0.3	8.6	4.5	109	0	NT	<1/5
85	<0.3	10.0	4.3	135	2	NT	<1/5
86	<0.3	13.1	4.7	39	5	NT	<1/5
87	<0.3	10	3.9	136	3	NT	<1/5
88	<0.3	4.2	6	137	5	NT	<1/5
89	<0.3	18.7	5.6	148	7	NT	<1/5
90	<0.3	8.1	5.2	216	3	NT	<1/5
91	<0.3	1.8	3.7	29	0	NT	<1/5
92	<0.3	5.5	4.2	68	6	NT	<1/5
93	<0.3	3.3	4.6	84	4	NT	<1/5
94	NT	0.4	4	20	0	NT	<1/5
95	NT	3	4.4	113	1	NT	<1/5
96	NT	5.8	4.1	82	0	NT	<1/5
97	NT	10	5.2	84	0	NT	<1/5
98	NT	12	4.8	174	4	NT	<1/5
99	NT	5	3.9	110	0	NT	<1/5
100	NT	4.5	3.7	97	0	NT	<1/5
101	NT	10.3	3.8	97	7	NT	<1/5
102	NT	2.7	3.9	102	1	NT	<1/5
103	NT	0.1	4	74	0	NT	<1/5
104	NT	3.6	4.1	107	0	NT	<1/5
105	NT	4.3	4.6	73	6	NT	<1/5
106	NT	8.9	3.9	33	10	NT	<1/5
107	NT	1.9	5.3	85	7	NT	<1/5
108	NT	10.7	4.9	127	0	NT	<1/5
109	NT	5.7	4.5	86	3	NT	<1/5
110	NT	4.6	5.2	151	0	NT	<1/5
111	NT	3	4.3	127	0	NT	<1/5
112	NT	2.1	4.4	17	1	NT	<1/5
113	NT	0.9	4.5	394	1	NT	<1/5
114	NT	0.0	4.3	26	3	NT	<1/5
115	NT	0.1	5.6	532	0	NT	<1/5
116	NT	12.4	4.8	143	2	NT	<1/5
117	NT	5.0	4.2	87	7	NT	<1/5
118	NT	4.3	4.6	68	2	NT	<1/5
Diagnostic test 2×2table	NT	Se: 86.15% (95%CI: 75.34–93.47) Sp: 100% (95%CI: 91.95–100)	Se: 89.39% (95%CI: 75.36–95.63) Sp: 100% (95%CI: 91.95–100)	Se: 100% (95%CI: 95–100) Sp: 100% (95%CI: 91.96–100)	Se: 100% (95%CI: 95–100) Sp: 100% (95%CI: 91.96–100)	NT	Se: 100% (95%CI: 94.87–100) Sp: 100% (95%CI: 91.96–100)
K cohen agreement vs. VNT	NT	K: 0.83 (95%CI: 0.73–0.93)	K: 0.87 (95%CI: 0.78–0.96)	K: 1 (95%: +/−0)	K: 1 (95%: +/−0)	NT	

**Table 2:** Quality values of the sequence data obtained.

		Name of Samples	
GenBank accession number	230860/12/20 (ISS81/21/3) Human 1-08-20 OL738656	240408/16/20 (ISS221/21/2) Mink 17-08-20 OL739154	350227/1/20 (ISS93/21/1) Mink 29-10-20 OL739160
Sequence quality parameters			
Coverage (cutoff > di 30×)	515×	1.072×	2.360×
Genome length (Ref. Acc. N° NC_045512.2; 29,993 bp)	29.826	29.612	29.835
Number of total reads	85.050	657.253	3.484.458
Number of mapped reads	83.850 (98.59%)	222.056 (33.7%)	392.713 (11.27%)

**Table 3:** Amino acid mutations observed in the Spike protein of the Italian human sequences available through GISAID (https://www.gisaid.org/, accessed on 30 May 2022) in 2020–2021.

			Spike Protein-Amino Acid Mutations							
	Collection date	Number of sequences	L216H	S477N	N501Y	D614G	P681H	Y453F	Mink Cluster V	Pango lin
Human sequence	1 August 2020	1	no	no	no	yes	no	no	no	B.1
Mink sequence	17 August 2020	1	yes	no	yes	yes	yes	no	no	B.1.1
Mink sequence	29 October 2020	1	no	yes	no	yes	no	no	no	B.1.160
Human sequences from Italy available in GISAID	2020	5.634	0%	230 (4.1%)	173 (3.1%)	5.343 (94.8%)	180 (3.2%)	no	no	
Human sequences from Italy available in GISAID	2021	90.147	0%	2.748 (3%)	33.363 (37%)	86.701 (96.2%)	32.622 (36.2%)	3 (0.003%)	no	

## Data Availability

Not applicable.

## Supplementary Materials

The following supporting information can be downloaded at: https://www.mdpi.com/article/10.3390/v14081738/s1, Figure S1: Amino acid sequence alignment of ACE2 from mink and human. Residues involved in the RBD-ACE2 interaction are identified with dots (Lan et al., 2020). Regions involved and residues required for interaction with the spike protein are underlined and shown in blue boxes, respectively.
