# Supplementary figures and images for: SARS-CoV-2 in a Mink Farm in Italy: Case Description, Molecular and Serological Diagnosis by Comparing Different Tests
Source: Viruses. 2022 Aug 8;14(8):1738. doi: 10.3390/v14081738 (PMC9415545; doi:10.3390/v14081738)

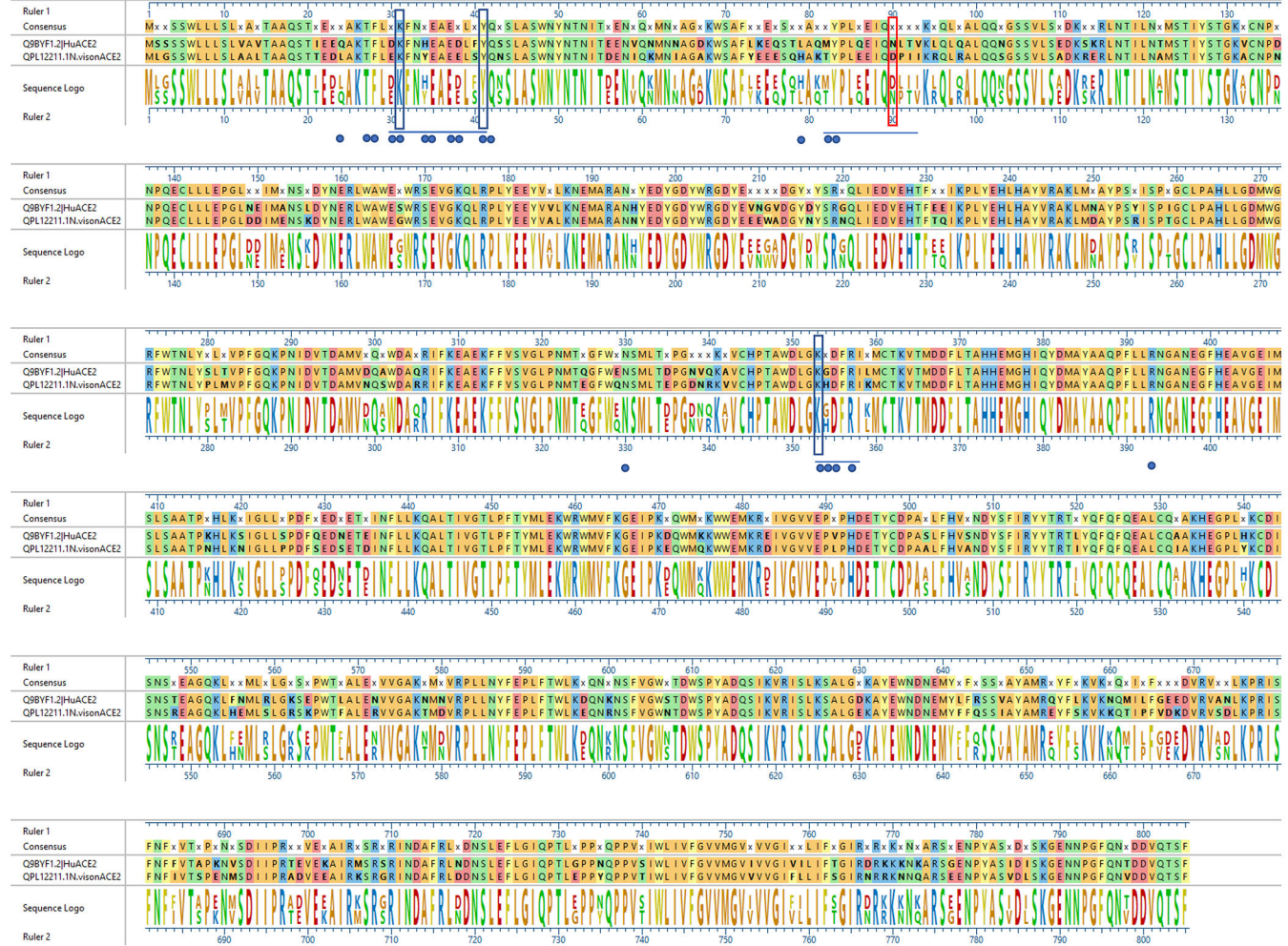

Supplement: Supplementary file 1 [file viruses-14-01738-s001.zip › viruses-1789393-supplementary.pdf]
